# Variations in ncRNA gene *LOC284889* and *MIF-794CATT* repeats are associated with malaria susceptibility in Indian populations

**DOI:** 10.1186/1475-2875-12-345

**Published:** 2013-09-25

**Authors:** Aditya N Jha, Pandarisamy Sundaravadivel, Sudhanshu S Pati, Pradeep K Patra, Kumarasamy Thangaraj

**Affiliations:** 1 CSIR - Centre for Cellular and Molecular Biology, Uppal Road, Hyderabad 500007, India; 2 Ispat General Hospital, Rourkela, Orrisa, India; 3 Pt. Jawaharlal Nehru Memorial Medical College, Raipur, Chhattisgarh, India

**Keywords:** Malaria, *MIF*, Non-coding RNA, Polymorphism, Indian populations, Diplotype

## Abstract

**Background:**

There are increasing evidences on the role of non-coding RNA (ncRNA) as key regulator of cellular homeostasis. *LOC284889* is an uncharacterized ncRNA gene on reverse strand to *MIF* mapped to 22q11.23. MIF, a lymphokine, regulates innate immune response by up-regulating the expression of *TLR4*, suppressing the p53 activity and has been shown to be involved in malaria pathogenesis.

**Methods:**

In this study, the possible effect of *MIF* variations on malaria susceptibility was investigated by re-sequencing the complete *MIF* gene along with 1 kb each of 5′ and 3′ region in 425 individuals from malaria endemic regions of the Orissa and Chhattisgarh states of India. The subjects comprised of 160 cases of severe malaria, 101 of mild malaria and 164 ethnically matched asymptomatic controls. Data were statistically compared between cases and controls for their possible association with *Plasmodium falciparum* malarial outcome.

**Results:**

It is the first study, which shows that the allele *A* (rs34383331*T > A*) in ncRNA is significantly associated with increased risk to *P. falciparum* malaria [severe: OR = 2.08, p = 0.002 and mild: OR = 2.09, P = 0.005]. In addition, it has been observed that the higher *MIF-794CATT* repeats (>5) increases malaria risk (OR = 1.61, p = 0.01). Further, diplotype (*MIF-794CATT* and rs34383331*T > A*) *5 T* confers protection to severe malaria (OR = 0.55, p = 0.002) while *6A* (OR = 3.07, p = 0.001) increases malaria risk.

**Conclusions:**

These findings support the involvement of ncRNA in malarial pathogenesis and further emphasize the complex genetic regulation of malaria outcome. In addition, the study shows that the higher *MIF-794CATT* repeats (>5) is a risk factor for severe malaria. The study would help in identifying people who are at higher risk to malaria and adapt strategies for prevention and treatment.

## Background

Malaria is one of the most common infectious disease, endemic in 104 countries [1], is caused by a protozoan parasites of the genus *Plasmodium*. The most serious form of disease is caused by *Plasmodium falciparum*. According to WHO [1], there were 219 million incidence of malaria in the year 2010 and an estimated 660,000 deaths worldwide. Malaria mortality is higher in children worldwide and malarial resistance genes serves as strongest know force for recent evolutionary selection in human genome, since first human started moving out of Africa [2]. Various population-specific natural genetic defense mechanisms have evolved in malaria-endemic regions [3], such as sickle cell trait, glucose-6-phosphate dehydrogenase deficiency, β-thalassaemia, duffy phenotypes; which are maintained in endemic populations by balancing selection [4,5]. The genetic basis of malaria resistance and susceptibility is complex in many ways as several genes have been found to be involved along with environmental and parasite genetic factors. Studies have confirmed that besides environmental factors and population diversity, polymorphisms in innate immunity genes such as Toll-like receptors (*TLR2, TLR4, TLR9*), chemokines, and cytokines as well as the heterogeneity in other immune-regulatory genes modulate malaria pathogenicity [6-11].

Among various effector molecules, cytokines plays crucial role as it speeds up the host inflammatory responses and coordinates the cell-mediated and humoral immune responses for the elimination and containment of invading microbes [12]. Failure of immune system to recognize invading pathogens at early stage, favours the unrestricted growth of microbes, which leads to potentially life threatening complications for the host. MIF (Macrophage migration inhibitory factor) is an important cytokine of host antimicrobial defense system, constitutively expresses with various other cytokines and promotes pro-inflammatory functions in both innate and acquired immunity [12,13]. MIF is produced mainly by T-cells as well as by monocytes, macrophages, dendritic cells, B cells, neutrophils and pituitary cells [14]. Conservation of *MIF* across species (chicken, fish, ticks, parasites, cyanobacteria and even in *Arabidopsis*) indicate its important biological functions [13]. Studies have documented the role of MIF in phosphorylation and activation of ERK1-ERK2-MAPK pathway (Extracellular-signal regulated kinase, Mitogen-activated protein kinase), cell proliferation, up-regulation of TLR4 expression and suppression of p53 and JAB1 (JUN-activation domain-binding protein 1) activity [15-20]. It has been suggested that MIF regulates pro-inflammatory innate immune response by up regulating the expression of TLR4 and suppressing the p53 activity and hence the prolonged cell survival [16,17,19].

Genetic variations in *MIF* have been shown to be associated with many infectious diseases, such as; sarcoidosis, malaria, schistosomiasis, trypanosomiasis, and leishmaniasis as well as inflammatory autoimmune diseases including rheumatoid arthritis, ulcerative colitis and atopy [12,14,21-28]. Two variations in the promoter region of *MIF*: *-173G/C* and −*794(CATT)* STR (short tandem repeat) regulate the expression of *MIF* and hence the serum level and disease susceptibility. In particular, allele *-173C* and higher *CATT* repeats (>5) are associated with higher MIF production [29-31], however, opposite patterns has also been reported in different populations, diseases and even cell types [32-34]. Studies on Zambian children with malaria demonstrated that higher *CATT* repeats (−794CATT*6/7/8) were correlated with increased parasitaemia, whereas -794CATT*5 was correlated with decrease in parasitaemia [35]. In line to this finding, study on Kenyan children demonstrated that higher STR repeats −*794(CATT)7-8* and *-173G* were associated with increased risk of severe malarial anaemia [32].

Non-coding RNA (ncRNA) acts as a key regulator of cellular homeostasis and modulates gene regulation (both in *cis* and *trans*), genome defense as well as chromosomal modifications. *LOC284889* is an uncharacterized gene located on the reverse strand to *MIF* mapped to genomic location 22q11.23. GRCh37/hg19 assembly of UCSC [36] and NCBI [37] have described it as an ncRNA gene while ensembl [38] as a putative protein coding. Dysregulations of ncRNA have been shown to involved in tumorigenesis; neurological, cardiovascular, developmental as well as various diseases [39-42].

In addition, several studies have demonstrated the functional significance of *MIF* gene polymorphism with malaria severity and pathogenesis in different ethnic populations across the globe [25,32,43-47]. However, to the best of knowledge no studies have documented the role of *MIF* gene polymorphism to the development of malaria in Indian populations. Study on Indian population is interesting as they have unique genetic makeup compared to rest of the world. Indian populations remain isolated for thousands of years hence accumulated unique set of mutations, which regulate the pathogenesis differently [8,9,48,49]. Therefore, objective of this study was to investigate the probable association of genetic variations in uncharacterized ncRNA gene *LOC284889* and *MIF* with *Plasmodium falciparum* malaria in well-characterized case–control groups.

## Methods

### Study subjects and sampling

This study consisted, a total of 425 malaria patients, which includes severe malaria (160), mild malaria (101) and asymptomatic control (164) (Table 1). About 5 ml of intra-venous blood samples were collected from the patients visiting/admitted for treatment for malaria at Ispat General Hospital, Rourkela, Orissa State and Pt. Jawaharlal Nehru Memorial Medical College, Raipur, Chhattisgarh State, India. These two states are among the *Plasmodium falciparum* endemic regions of India having similar climatic conditions. All individuals representing the malaria cohort were clinically characterized as per WHO guidelines [50] and as described previously [8,9]. Samples were *Plasmodium falciparum* positive and the microscopic result were re-validated by polymerase chain reaction (PCR) using species specific-primers; targeting conserved 18S rRNA gene of the parasite [51]. Ethnically matched asymptomatic control samples were collected from the same malaria endemic region. The controls were not having any clinical symptoms at the time of sampling. Blood samples were collected from remote areas, making it difficult to have a long-term follow-up.

**Table 1:** Characteristics of studied subjects segregated according to clinical classification

	**Sample size**	**Mean age (year) ± SD**	**Male: Female**
Severe malaria	160	27.55 ± 12.34	92:68
Mild malaria	101	30.24 ± 15.71	67:34
Asymptomatic control	164	29.87 ± 19.53	92:72

### Consent and ethical committee approval

An informed written consent was obtained from all the individuals. The study was approved by the Institutional Ethical Committee (IEC) of Centre for Cellular and Molecular Biology, Hyderabad, India; Ispat General Hospital, Rourkela, Orissa, India; and Pt. Jawaharlal Nehru Memorial Medical College, Raipur, Chhattisgarh, India.

### Genomic DNA isolation and primer designing

Genomic DNA was extracted from whole blood using the protocol described previously [52]. The reference sequence was retrieved from ENSEMBL [38]. Target specific primer pairs were designed using the Primer-BLAST [53], MacVector and the Amplify 3X software [54].

### Gene sequencing

The 3 kb of target DNA including 1 kb each of upstream and downstream region was PCR amplified. The primer pairs used for the amplification have been given in Table 2. Amplifications were performed using Takara EmeraldAmp GT PCR kit on GeneAmp 9700 (Applied Biosystem) and Thermal Cycler (Eppendorf) following manufacturer’s protocol. Thermal cycling parameters for amplification were: initial denaturation at 94°C for 5 minute, followed by 35 cycles of 30 second at 94°C, 25 second at 62 - 67°C (Table 2) and 1 minute 30 second at 72°C for extension, followed by a final extension of 5 minute at 72°C. PCR products were cleaned up using 2 μl of Exo-SAP (USB, Affymetrix) to 5 μl of PCR product, incubating at 37°C for 20 minute followed by enzyme deactivation at 80°C for 15 minutes. After Exo-Sap treatment, 1 μl of the purified products were used as templates for sequencing, using the Big-Dye terminator (v. 3.1) cycle sequencing kit (Applied Bio systems) on an ABI 3730XL DNA sequencer, according to the manufacturer’s instructions. Sequencing was done with a total of five sets of primers as listed in Table 2. DNA variations were identified after assembling with the reference sequence using Auto-Assembler software (Applied Bio system). Observed variations were validated by re-sequencing in a subset of samples.

**Table 2:** **Primer sequences and conditions for the PCR of the *MIF *gene**

**PCR set**	**Primer locations**	**Primer sequences (5**′ **- 3**′**)**	**Annealing temperature (°C)**	**PCR product size**
SET 1	MIF-F (−1202)	GAGCAGTGGACACTCAGTCAGC	65	600 bp
	MIF-R (−647)	CCTCTGGGCAACTTCAGCTCCT		
SET 2	MIF-F (−740)	GCACCTGCTAGATGGTCCCCG	65	696 bp
	MIF-R (−86)	AGTGGGGAAGTCACCGCCTG		
SET 3	MIF-F (−315)	TTCATCTCTGGAAGGGTAAGGGG	62	1370 bp
	MIF-R(+1009)	GACACTGGGGCTCCTCTGTTAGG		
	MIF-Internal	AGTGGTGTCCGAGAAGTCAG		
SET 4	MIF-F (+747)	TAAGAGCCGCAGGGACCCAC	67	596 bp
	MIF-R (+1302)	TGGCAGTGAGTGGCTCTGGG		
SET 5	MIF-F (+1182)	GGGAGGAGGAGTTGGAGTTGGG	65	600 bp
	MIF-R (+1757)	CCCTGGAGCTTCTATTCTCCTTCCT		

Primer locations are relative to transcription start site.

### Statistical analysis

Allele and genotype frequencies were analysed by simple gene counting and expectation-maximum (EM) algorithm and the significance of deviations from Hardy-Weinberg equilibrium was tested using plink v1.7 [55]. The allele and genotype distribution and test of association were performed using plink v1.7. Fisher’s two tailed exact test and logistic regression were performed by SPSS software (ver. 20). Linkage disequilibrium (LD) analysis was performed using Haploview v4.2 software [56]. Chi-square contingency-table test results were interpreted by standardized residual method of post-hoc analysis [57]. The haplotype-based association test for multiallelic markers was performed using WHAP v2.09 package [58]. In all analysis, a two tailed p-value less than 0.05 were considered significant.

## Results

### *MIF* variations and *P. falciparum* malaria

**Figure 1:**
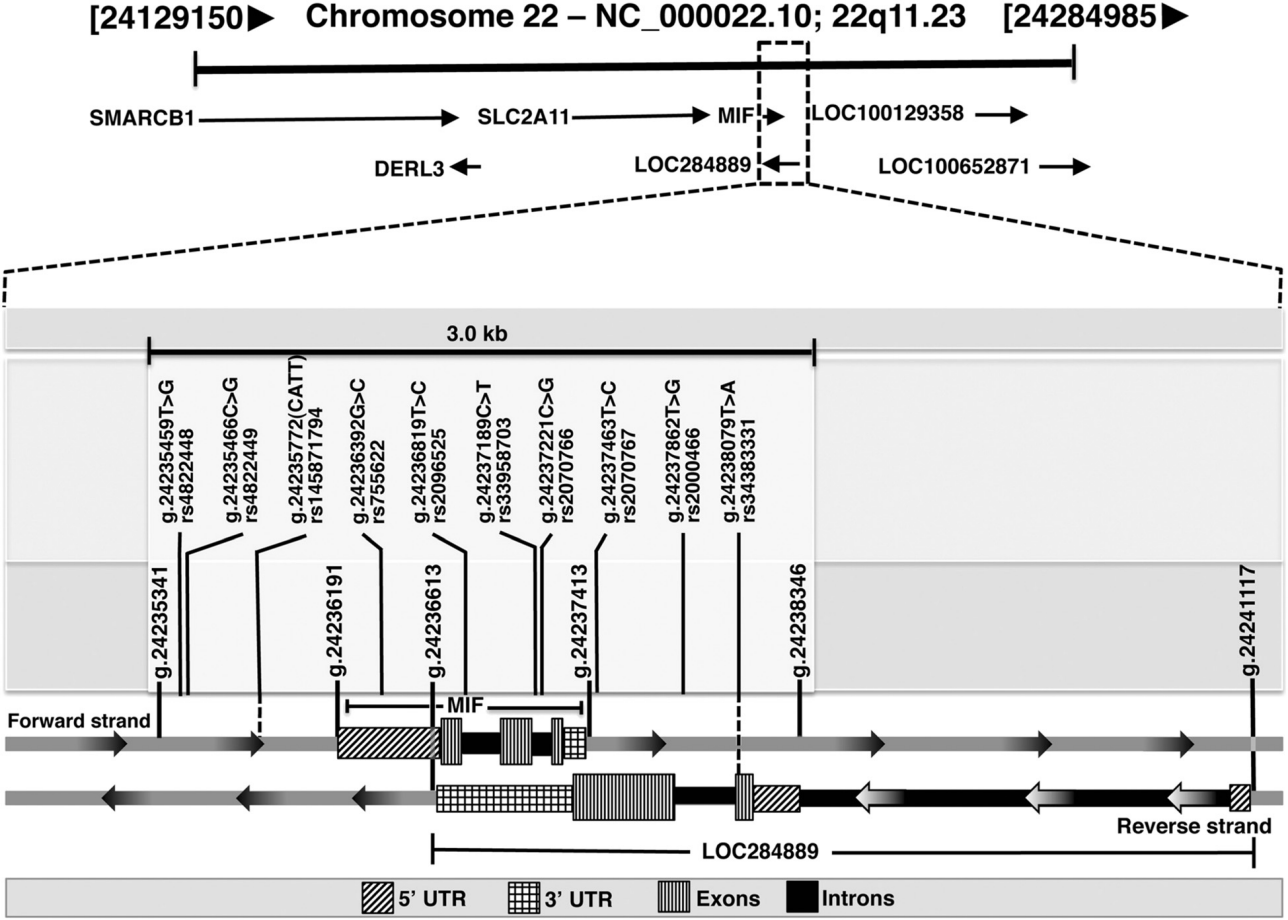
**Genomic map of *MIF *and ncRNA gene *LOC284889*.** The genomic coordinates are as per ensembl genomic assembly GRCh37. The observed variations have been placed proportionately on the map.

**Figure 2:**
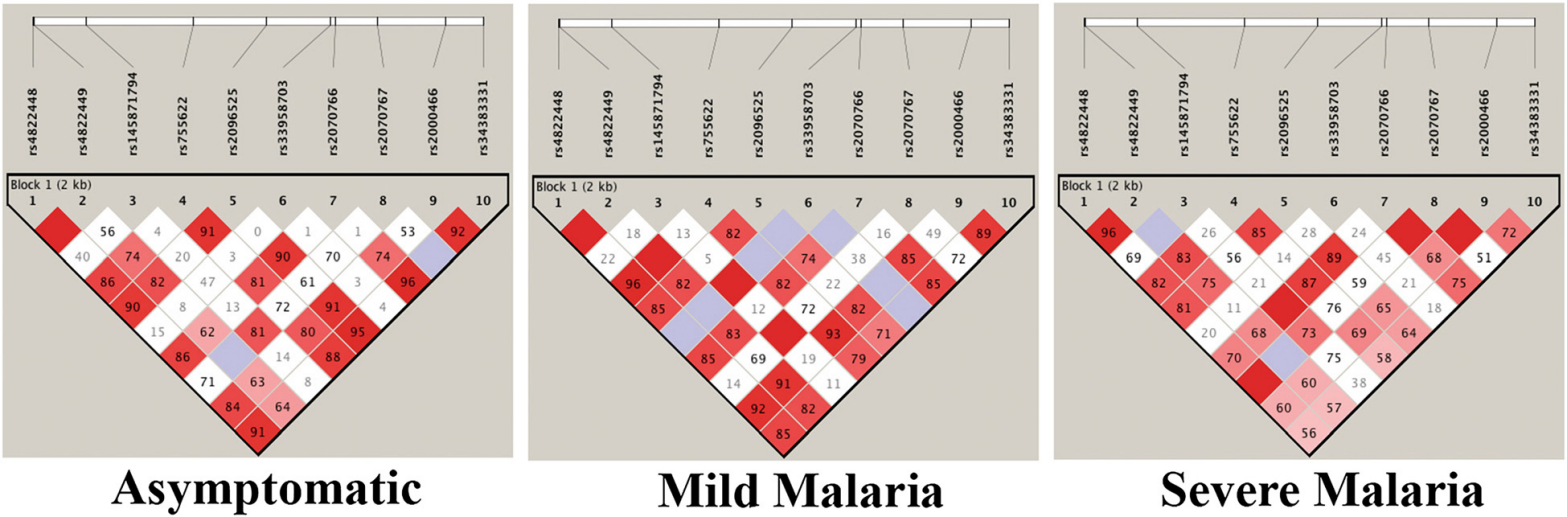
**Linkage Disequilibrium pattern of *MIF *variants in asymptomatic, mild and severe malaria groups.** Empty squares indicate a high degree of LD (D’ = 1). Numbers indicate the D’ value expressed as a percentile.

Sequencing of *MIF* gene along with 1 kb each of upstream and downstream lead to detection of a total of nine single nucleotide polymorphism (SNPs) and one STR *CATT* repeat at −794 position (Additional file 1). All the variations were in Hardy Weinberg equilibrium. Genomic context of *MIF* and *LOC284889* along with observed variations and their genomic coordinates as per Ensembl Grch37 [38] and NCBI [37] have been shown in Figure 1. The LD pattern of these variations was stronger in asymptomatic and mild groups compared to severe malaria group (Figure 2). Although, four alleles of the *-794CATT* STR have been reported [24,32], this study observed only three alleles [*(CATT)5*, *(CATT)6* and *(CATT)7*] in Indian population. Previous studies have demonstrated that the increase in the *CATT* repeats beyond five lead to the increase in MIF level [24,29]. Therefore, further analysis was done by down-coding the tri-allelic STR polymorphism as a biallelic: (1) *(CATT)5* and (2) *(CATT)6-7*; by pooling *(CATT)6* and *(CATT)7* as one group (> 5 repeat). The distribution of *MIF* genotype and allele frequencies in all study groups (asymptomatic, mild and severe malarial cases) has been summarized in Additional file 1. The genotype and allele frequencies of *CATT* repeat at −794 (rs145871794) differed significantly between severe malaria and asymptomatic control (genotype: χ^2^_2_ = 7.24, p = 0.02; allele: χ^2^_1_ = 6.59, p = 0.01, OR = 0.62, 95% CI = 0.43 - 0.89). However, in present study, no statistically significant differences were observed either in genotype frequency distribution (χ^2^_2_ = 2.78, p = 0.2) or in allele frequency distribution (χ^2^_1_ = 1.53, p = 0.2, OR = 0.77 95% CI = 0.52 - 1.26) between asymptomatic and mild malaria (Additional file 1).

### ncRNA (*LOC284889*) gene polymorphism rs34383331 and *P. falciparum* malaria

An uncharacterized ncRNA gene (*LOC284889*) exists on the opposite strand to *MIF* (Figure 1). A *T > A* transversion (rs34383331) was observed in ncRNA within 1 kb downstream region of *MIF*. The genotype frequency of the observed variation rs34383331 differed significantly among the case–control groups (severe: χ^2^_2_ = 8.6, p = 0.0133; mild: χ^2^_2_ = 8.6, p = 0.0133). The allele frequency also differed significantly among these groups (severe: OR = 2.076, 95% CI = 1.30 – 3.32, p = 0.002; mild: OR = 2.078, 95% CI = 1.24 – 3.48, p = 0.005) (Additional file 1). The post-hoc analysis of chi-square contingency table showed over-representation of genotype *TT* (82.9%, z-score = 3.3) and under-representation of genotype *TA* in asymptomatic group (15.2%, z-score = −2.9) compared to both severe and mild malaria groups. These findings assign a protective role to *T* allele.

### *MIF-794 CATT* repeat length polymorphism and malaria severity

**Figure 3:**
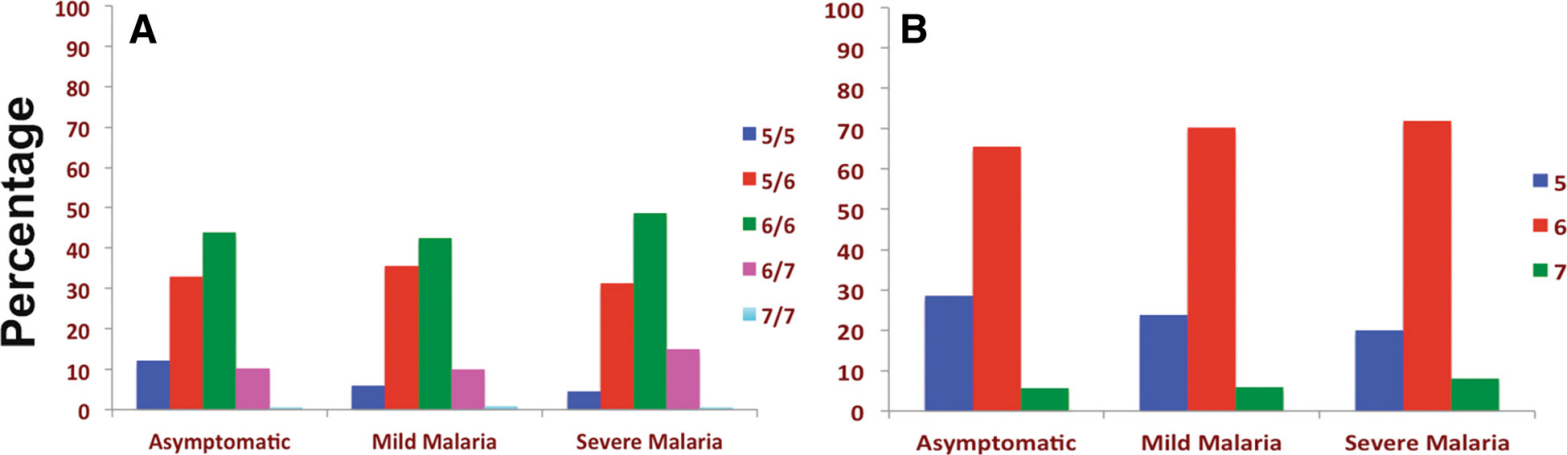
**Distribution of *MIF -794CATT *STR repeats in case–control groups. A**. Genotype and **B**. allele.

**Figure 4:**
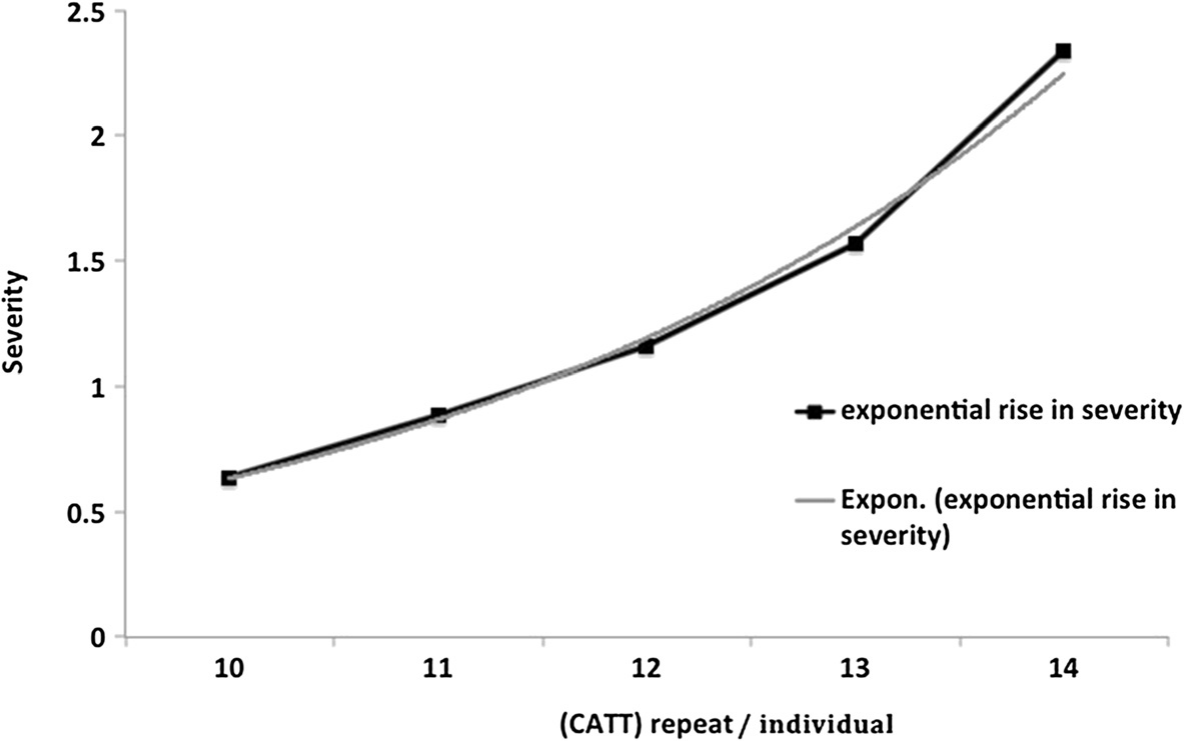
**Odds of developing severe malaria per unit rise of (CATT) repeats.** The malaria severity increases exponentially following the trend log_e_(severity) = [−3.45 + (0.3 ± 0.13) (number of CATT)] [Trend line: logit(P) = log_e_(odds) = log_e_(p/q) = a + bX].

Further, investigation showed the association between *-794CATT* tetra-nucleotide repeats number variation and malaria severity. The *-794CATT* genotypes (CATT*5/5, CATT*5/6, CATT*6/6, CATT*6/7, and CATT*7/7) frequency distributions and statistical comparisons have been shown in Figure 3 and Table 3. The frequency of genotype -794CATT*5/5 was significantly lower in severe malaria (OR = 0.33, 95% CI = 0.13 – 0.80, p = 0.015), however, there were no significant difference between mild malaria and asymptomatic control. The allelic distribution show that the allele -794CATT*5 was significantly under represented in severe malaria (OR = 0.62, 95% CI = 0.43 – 0.89, p = 0.011). In line of these findings, higher *CATT* repeats (CATT*6/*7) were observed significantly higher in severe malaria group (OR = 1.61, 95% CI = 1.12 – 2.31, p = 0.01) inferring higher number of *CATT* repeat (CATT*6/*7) influence the susceptibility to malaria (Table 3). The logistic regression analysis show the severity of malaria increases exponentially with per unit rise of *CATT* repeats (χ^2^_1_ = 4.76, p = 0.029, OR = 1.34, 95% CI = 1.03 – 1.75) (Figure 4).

**Table 3:** **Comparison of *MIF -794CATT *STR genotype and allele frequencies among case–control groups**

**Genotype**	**Asymptomatic (n = 164)**	**Mild (n = 101)**	**Severe (n = 160)**	**Asymptomatic vs. mild malaria#**		**Asymptomatic vs. severe malaria#**	
***−794 CATT***				**P-value**	**OR (95% CI)**	**P-value**	**OR (95% CI)**
5/5	20 (12.1)	6 (5.9)	7 (4.4)	NS	---	0.015	0.33 (0.13 – 0.80)
5/6	54 (32.9)	36 (35.6)	50 (31.2)	NS	---	NS	---
6/6	72 (43.9)	48 (42.5)	78 (48.7)	NS	---	NS	---
6/7	17 (10.3)	10 (9.9)	24 (15.0)	NS	---	NS	---
7/7	1 (0.6)	1 (0.9)	1 (0.6)	NS	---	NS	---
**Allele**	**Asymptomatic (n = 328)**	**Mild (n = 202)**	**Severe (n = 320)**	**P-value**	**OR (95% CI)**	**P-value**	**OR (95% CI)**
5	94 (28.5)	48 (23.7)	64 (20.0)	NS	---	0.011	0.62 (0.43 – 0.89)
6	215 (65.5)	142 (70.2)	230 (71.9)	NS	---	NS	---
7	19 (5.79)	12 (5.9)	26 (8.1)	NS	---	NS	---
grouped							
5	94 (28.5)	48 (23.7)	64 (20.0)	NS		0.01	0.62 (0.43 – 0.89)
> 5	234 (71.3)	154 (76.2)	256(80.0)	NS	---	0.01	1.61 (1.12 – 2.31)

*NS*: Not Significant; # Fisher’s two tailed exact test; Values in parenthesis have been rounded off.

### Diplotype (−*794CATT* and rs34383331*T > A*) and malaria severity

Earlier studies demonstrated that the MIF serum level is modulated by two promoter polymorphisms *-794CATT* and *-173G/C*[24,29,32]. However, this study did not observe any association with *-173G/C*. Instead, downstream polymorphism rs34383331 *T > A* observed in ncRNA was associated significantly with malaria. Therefore, these two variations (−*794CATT* and rs34383331*T > A*) were considered for diplotype reconstruction and analysed for their possible association with malaria (Table 4). The diplotype *6A* was observed significantly higher in mild malaria group (OR = 2.66, 95% CI = 1.29 – 5.48, p = 0.018) [Bonferroni corrected p-value] compared to asymptomatic control. However, diplotype *5 T* was significantly lower (OR = 0.55, 95% CI = 0.38 – 0.80, p = 0.002), while diplotype *6A* (OR = 3.07, 95% CI = 1.59 – 5.91, p = 0.002) [Bonferroni corrected p-value] was significantly higher in severe malaria compared to the asymptomatic control (Table 4).

**Table 4:** **Distribution and comparison of *MIF *diplotypes (*MIF -794CATT *and rs34383331*T > A) *in case–control groups**

**Haplotype**	**Asymptomatic (%) (Total = 328)**	**Mild (%) (Total = 202)**	**Severe (%) (Total = 320)**	Asymptomatic* vs. mild malaria		Asymptomatic* vs. severe malaria	
**MIF-794 and rs34383331**				**P - value**	**OR (95% CI)**	**P - value**	**OR (95% CI)**
6 T	202 (61.6)	122 (60.4)	194 (60.6)	NS	---	NS	---
5 T	90 (27.4)	42 (20.8)	55 (17.2)	NS	---	0.002	0.55 (0.38 – 0.80)
7A	14 (4.3)	10 (4.9)	12 (3.7)	NS	---	NS	---
6A	13 (4)	20 (9.9)	36 (11.2)	0.018	2.66 (1.29 – 5.48)	0.002	3.07 (1.59 – 5.91)
7 T	5 (1.5)	2 (1)	14 (4.4)	NS	---	NS	---
5A	4 (1.2)	6 (2.97)	9 (2.8)	NS	---	NS	---

*NS* Not Significant, *Fisher’s two tailed exact test; Values in parenthesis have been rounded off; P - value was corrected for multiple comparisons by Bonferroni’s method.

## Discussion

Genetic variations play an important role in the occurrence and development of malaria and its severity. Variations in several host genes (*HBB*, *IL4*, *IL12*, *TNF*, *LTA*, *NCR3* and *FCGR2A*) have been reported to be associated with malaria outcome [59]. Therefore, studies on relationship between gene polymorphism and malaria susceptibility are of greater interest in the prevention and control of malaria.

MIF is a lymphokine involved in cell-mediated immunity, immune-regulation, and inflammation. MIF plays a role in the regulation of macrophage function in host defense through the suppression of anti-inflammatory effects of glucocorticoids. Further, MIF regulates pro-inflammatory innate immune response by up-regulating the expression of TLR4 and suppressing the p53 activity [16,17,19]. Several studies have shown that the *MIF* genetic variants regulate the MIF serum levels in various diseases, such as; sarcoidosis, malaria, schistosomiasis, trypanosomiasis, leishmaniasis, rheumatoid arthritis, ulcerative colitis and atopy [12,14,21-28]. Further, its clinical significance also varies in different populations [25,32,43-47]. Additionally, studies have documented that two *MIF* promoter polymorphism *-794CATT* repeat and *-173G/C* regulates the level of *MIF* expression and alter the serum level of MIF; particulary an increase in number of *-794CATT* STR repeats lead to elevated levels of *MIF* expression [29-31]. Studies on Indian populations have shown association between elevated level of MIF in the peripheral blood of cerebral malarial with the fatal outcome [47]. A recent study also has demonstrated association between elevated level of inter-villous MIF level with still birth and low birth deliveries in Central Indian population [60]. However, both of these studies have not looked into the *MIF* polymorphism. To the best of the knowledge, no study to date has investigated the association of *MIF* polymorphism with malarial susceptibility in Indian populations. Therefore, this study investigated the possible association between *MIF* gene polymorphism and *P. falciparum* malaria outcome in a well-defined ethnically matched case–control cohort from Orissa and Chhattisgarh, the malaria endemic states of India.

The present study observed significant difference in genotype and allele distribution of *MIF* gene among ethnically matched case–control groups. The CATT*5 was significantly higher in asymptomatic control, while CATT*6 and CATT*7 were significantly higher in malaria groups suggesting that the higher repeat as the risk factor to severe malaria in Indian populations. This finding is in agreement with the earlier studies, which have demonstrated that higher repeats increases susceptibility to malaria [29,32,35,47]. Previous study has demonstrated that the expansion of *-794CATT* repeat is directly proportionate to the severity of malaria among the children of Zambia and Kenya [32,35]. Although the present study shows the association of higher *-794CATT* repeats with the severity of malaria in Indian adults, similar association in children cannot be ignored.

Further, earlier study has also documented a strong LD between *-794CATT* repeat and *-173G/C* as well as association of *-173G/C* with severe malarial anaemia in African populations [32]. However, this study did not observe strong LD between these two promoter polymorphisms (Figure 2). In addition, *-173G/C* also did not show any association with malaria in Indian population. This observations is not surprising as similar heterogeneity have been observed among Indian populations due to their unique genetic architecture and they show varied response to pathogen and other diseases [8,9,48,49].

Interestingly, this is the first study which shows that the genotype and allele distribution of rs34383331*T > A,* present in an uncharacterized ncRNA (*LOC284889*) and located on the reverse strand to *MIF*, differ significantly between malaria cases and asymptomatic control groups. Further, this SNP is in strong LD with promoter as well as ORF variants in asymptomatic and mild malaria group compared to the severe malaria group. The genotype *TT* was significantly higher while genotype *TA* was significantly lower in asymptomatic control compared to malaria groups, which assigns a protective role of allele *T* against malaria.

Further, the diplotype reconstruction of promoter variant *-794CATT* and ncRNA variant rs34383331 *T>A* show that the diplotype *5 T* is significantly lower in severe malaria, while *6A* were higher in severe malaria compared to asymptomatic control. This again elucidates that the individuals with additional *CATT* repeat and allele *A* have high risk for malaria.

## Conclusions

In conclusion, for the first time, this study reports the association of an ncRNA with malaria pathogenesis. In addition, this study also observed that the *MIF* polymorphism is associated with malaria pathogenesis in Orissa and Chhattisgarh populations of India. These findings, which show the involvement of ncRNA along with previous studies on *IL4* and *IFNB*[8,9], further emphasize the complex genetic regulation of malaria outcome. Genotyping this polymorphism in further larger case–control and cohort studies as well as on ethnically different populations is strongly recommended for the better estimation of malaria risk associated with this polymorphism. This study also emphasizes the role of host genetics in modulating pathogenesis and disease outcome. As the malaria severity is the outcome of complex genetic interplay; numerous variants are likely to act in tandem. Therefore, the studies of down-stream as well as other immune-regulator genes are equally important to understand the molecular basis of disease severity.

## Competing interests

The authors declare that they do not have any conflict of interest or competing / financial interests.

## Authors’ contributions

ANJ and KT planned and designed the experiment. SSP and PKP collected patients blood sample and carried out clinical assessment. ANJ and PS prepared samples and performed experiments. ANJ analysed the results. ANJ and PS prepared the manuscript. ANJ and KT prepared the final manuscript. All authors read and approved the final manuscript.

## Supplementary Material

Additional file 1 **Genotype and allelic distribution of *MIF *variants in case–control groups.** Click here for file
