# Supplementary material for: Variations in ncRNA gene LOC284889 and MIF-794CATT repeats are associated with malaria susceptibility in Indian populations
Source: Malar J. 2013 Sep 25;12:345. doi: 10.1186/1475-2875-12-345 (PMC3849407; doi:10.1186/1475-2875-12-345)
Supplement: Additional file 1 — Genotype and allelic distribution of MIF variants in case–control groups. [file 1475-2875-12-345-S1.pdf]

Variations in ncRNA gene *LOC284889* and *MIF -794CATT* repeats are associated with malaria susceptibility in Indian populations

Aditya Nath Jha[1#], Pandarisamy Sundaravadivel[1#], Sudhanshu S Pati[2], Pradeep K Patra[3], Kumarasamy Thangaraj[1*]

Additional File 1: Genotype and allele distribution of *MIF* variants in case-control groups.

Group	Genotype (%)			Genotype Comparison		Allele (%)		Allele Comparison		
				$^{@}\chi^2_2$	P-value			$^{\&}\chi^2_1$	P-value	OR (95% CI)
**rs48822448 T>G**	**TT**	**TG**	**GG**			**T**	**G**			
Asymptomatic (n=164)	115(70.1)	43(26.2)	6(3.6)			273(83.2)	55(16.7)			
Mild (n = 101)	68 (67.3)	32 (31.6)	1(0.9)	2.42	0.30	168 (83.1)	34 (16.8)	0.0003	0.98	1.01 (0.63 – 1.61)
Severe (n = 160)	109 (68.1)	45 (28.1)	6 (3.7)	0.16	0.92	263 (82.1)	57 (17.8)	0.12	0.72	1.08 (0.72 – 1.62)
Mild vs. Severe	---	---	---	2.03	0.36	---	---	0.08	0.78	0.93 (0.58 – 1.49)
**rs4822449 C>G**	**CC**	**CG**	**GG**			**C**	**G**			
Asymptomatic (n=164)	127 (77.4)	32 (19.5)	5 (3.0)			286 (87.1)	42(12.8)			
Mild (n = 101)	72 (71.2)	28 (27.7)	1 (0.9)	3.35	0.19	172 (85.1)	30 (14.8)	0.45	0.50	1.19 (0.72 – 1.97)
Severe (n = 160)	126 (78.7)	28 (17.5)	6 (3.75)	0.31	0.86	280 (87.5)	40 (12.5)	0.014	0.91	0.97 (0.61 – 1.55)
Mild vs. Severe	---	---	---	5.23	0.07	---	---	0.59	0.44	1.22 (0.73 – 2.03)
**rs145871794 (-794CATT*5/6/7)**	**(CATT)6-7,6-7**	**(CATT)5,6-7**	**(CATT)5,5**			**(CATT)>5**	**(CATT)5**			
Asymptomatic (n=164)	90(46.7)	54(39.0)	20 (13.4)			234(71.3)	94 (28.6)			
Mild (n = 101)	59(48.5)	36(44.5)	6 (6.9)	2.77	0.25	154 (76.2)	48 (23.7)	1.53	0.21	1.29 (0.86 – 1.93)
Severe (n = 160)	103(64.4)	50(31.2)	7 (4.4)	7.24	0.03	256 (80.0)	64 (20.0)	6.59	0.01	0.62 (0.43 – 0.89)
Mild vs. Severe	---	---	---	1.02	0.60	---	---	1.04	0.31	1.25 (0.82 – 1.91)
**rs755622 G>C (-173)**	**GG**	**GC**	**CC**			**G**	**C**			
Asymptomatic (n=164)	123 (75.0)	27 (16.4)	4 (2.4)			283 (86.2)	45 (13.7)			
Mild (n = 101)	67 (66.3)	32 (31.6)	2 (1.9)	2.71	0.26	166 (82.1)	36 (17.8)	1.62	0.20	1.36 (0.84 – 2.20)
Severe (n = 160)	107 (66.8)	45 (28.1)	8 (5)	3.18	0.20	259 (80.9)	61 (19.0)	3.38	0.07	1.48 (0.97 – 2.25)
Mild vs. Severe	---	---	---	1.74	0.49	---	---	0.13	0.72	0.92 (0.58 – 1.45)

**rs2096525 T>C**	**TT**	**TC**	**CC**			**T**	**C**			
Asymptomatic (n=164)	118 (71.9)	42 (25.6)	4 (2.4)			278 (84.7)	50 (15.2)			
Mild (n = 101)	67 (66.3)	32 (31.6)	2 (1.9)	1.17	0.56	166 (82.1)	36 (17.8)	0.61	0.43	1.21 (0.75 – 1.93)
Severe (n = 160)	102 (63.7)	50 (31.2)	8 (5.0)	3.14	0.21	254 (79.3)	66 (20.6)	3.14	0.07	1.44 (0.96 – 2.17)
Mild vs. Severe	---	---	---	1.54	0.46	---	---	0.62	0.43	0.83 (0.53 – 1.31)
**rs33958703 C>T**	**CC**	**CT**	**TT**			**C**	**T**			
Asymptomatic (n=164)	150 (91.4)	12 (7.3)	2 (1.2)			312 (95.1)	16 (4.8)			
Mild (n = 101)	91 (90.0)	10 (9.9)	0 (0.0)	1.75	0.42	192 (95.0)	10 (4.9)	0.001	0.97	1.02 (0.45 – 2.28)
Severe (n = 160)	142 (88.7)	12 (7.5)	6 (3.7)	2.17	0.34	296 (92.5)	24 (7.5)	1.92	0.16	1.58 (0.82 – 3.03)
Mild vs. Severe	---	---	---	4.22	0.12	---	---	1.32	0.25	0.64 (0.30 – 1.37)
**rs2070766 C>G**	**CC**	**CG**	**G**			**C**	**G**			
Asymptomatic (n=164)	130 (79.2)	30 (18.2)	4 (2.4)			290 (88.4)	38 (11.5)			
Mild (n = 101)	71 (70.2)	25 (24.7)	5 (4.9)	3.08	0.21	167 (82.6)	35 (17.3)	3.47	0.06	1.56 (0.97 – 2.63)
Severe (n = 160)	115 (71.8)	38 (23.7)	7 (4.3)	2.69	0.27	268 (83.7)	52 (16.2)	2.95	0.08	1.48 (0.94 – 2.32)
Mild vs. Severe	---	---	---	0.09	0.95	---	---	0.1	0.75	1.08 (0.67 – 1.72)
**rs2070767 T>C**	**TT**	**TC**	**CC**			**T**	**C**			
Asymptomatic (n=164)	104 (63.4)	53 (32.3)	7 (4.2)			261 (79.5)	67 (20.4)			
Mild (n = 101)	65 (64.3)	35 (34.6)	1 (0.9)	2.34	0.32	165 (81.6)	37 (18.3)	0.35	0.55	0.87 (0.56 – 1.37)
Severe (n = 160)	111 (69.3)	41 (25.6)	8 (5.0)	1.78	0.41	263 (82.1)	57 (17.8)	0.71	0.38	0.84 (0.57 – 1.25)
Mild vs. Severe	---	---	---	4.85	0.09	---	---	0.02	0.88	1.03 (0.65 – 1.63)
**rs2000466 T>G**	**TT**	**TG**	**GG**			**T**	**G**			
Asymptomatic (n=164)	124 (75.6)	38 (23.1)	2 (1.2)			284 (86.5)	42 (12.4)			
Mild (n = 101)	67 (66.3)	31 (30.6)	3 (2.9)	3.12	0.21	165 (81.6)	37 (18.3)	2.99	0.08	1.53 (0.94 – 2.47)
Severe (n = 160)	111 (69.3)	42 (26.2)	7 (4.3)	3.65	0.16	264 (82.5)	56 (17.5)	2.78	0.09	1.44 (0.94 – 2.23)
Mild vs. Severe	---	---	---	0.84	0.66	---	---	0.06	0.81	1.06 (0.67 – 1.67)
**rs34383331 T>A**	**TT**	**TA**	**AA**			**T**	**A**			
Asymptomatic (n=164)	136 (82.9)	25 (15.2)	3 (1.8)			297 (90.5)	31 (9.4)			
Mild (n = 101)	68 (67.3)	30 (29.7)	3 (2.9)	8.63	0.0134	166 (82.1)	36 (17.8)	7.93	0.0048	2.08 (1.24 – 3.48)

Severe (n = 160)	111 (69.3)	41 (25.6)	8 (5.0)	8.63	0.0133	263 (82.1)	57 (17.8)	9.65	0.0018	2.07 (1.30 – 3.32)
Mild vs. Severe	---	---	---	1.02	0.60	---	---	0.01	0.92	1.00 (0.63 – 1.59)

* Tri-allelic CATT repeats were down coded to bi-allelic as $(CATT)_5$ and $(CATT)_{6-7;}$ $: ncRNA (LOC284889) polymorphism; Data were compared between asymptomatic controls and each case group using chi square test. @2x3 contingency chi square test; &2x2 contingency chi square test; Values in parenthesis have been rounded off;
